# Phosphodiesterase III inhibitor promotes drainage of cerebrovascular β-amyloid

**DOI:** 10.1002/acn3.79

**Published:** 2014-07-08

**Authors:** Takakuni Maki, Yoko Okamoto, Roxana O Carare, Yoshiki Hase, Yorito Hattori, Cheryl A Hawkes, Satoshi Saito, Yumi Yamamoto, Yasukazu Terasaki, Hatsue Ishibashi-Ueda, Akihiko Taguchi, Ryosuke Takahashi, Taihei Miyakawa, Raj N Kalaria, Eng H Lo, Ken Arai, Masafumi Ihara

**Affiliations:** 1 Department of Neurology, Graduate School of Medicine, Kyoto UniversityKyoto, Japan; 2 Departments of Radiology and Neurology, Massachusetts General Hospital and Harvard Medical SchoolCharlestown, Massachusetts; 3 Department of Pathology, National Cerebral and Cardiovascular CenterOsaka, Japan; 4 Division of Clinical Neurosciences, Southampton General Hospital, Southampton UniversityHampshire, United Kingdom; 5 Department of Regenerative Medicine and Tissue Engineering, National Cerebral and Cardiovascular CenterOsaka, Japan; 6 Department of Regenerative Medicine Research, Institute of Biomedical Research and InnovationKobe, Japan; 7 Amakusa HospitalKumamoto, Japan; 8 Institute for Ageing and Health, NIHR Biomedical Research Building, Newcastle University, Campus for Ageing and VitalityNewcastle upon Tyne, United Kingdom; 9 Department of Stroke and Cerebrovascular Diseases, National Cerebral and Cardiovascular CenterOsaka, Japan

## Abstract

**Objective:**

Brain amyloidosis is a key feature of Alzheimer's disease (AD). It also incorporates cerebrovascular amyloid β (Aβ) in the form of cerebral amyloid angiopathy (CAA) involving neurovascular dysfunction. We have recently shown by retrospective analysis that patients with mild cognitive impairment receiving a vasoactive drug cilostazol, a selective inhibitor of phosphodiesterase (PDE) III, exhibit significantly reduced cognitive decline. Here, we tested whether cilostazol protects against the disruption of the neurovascular unit and facilitates the arterial pulsation-driven perivascular drainage of Aβ in AD/CAA.

**Methods:**

We explored the expression of PDE III in postmortem human brain tissue followed by a series of experiments examining the effects of cilostazol on Aβ metabolism in transgenic mice (Tg-SwDI mice) as a model of cerebrovascular β-amyloidosis, as well as cultured neurons.

**Results:**

We established that PDE III is abnormally upregulated in cerebral blood vessels of AD and CAA subjects and closely correlates with vascular amyloid burden. Furthermore, we demonstrated that cilostazol treatment maintained cerebral hyperemic and vasodilative responses to hypercapnia and acetylcholine, suppressed degeneration of pericytes and vascular smooth muscle cells, promoted perivascular drainage of soluble fluorescent Aβ_1-40_, and rescued cognitive deficits in Tg-SwDI mice. Although cilostazol decreased endogenous Aβ production in cultured neurons, C-terminal fragment of amyloid precursor protein expression was not altered in cilostazol-treated Tg-SwDI mice.

**Interpretation:**

The predominant action of cilostazol on Aβ metabolism is likely to facilitate Aβ clearance due to the sustained cerebrovascular function in vivo. Our findings mechanistically demonstrate that cilostazol is a promising therapeutic approach for AD and CAA.

## Introduction

Alzheimer's disease (AD) is the most common form of dementia. In addition to neurofibrillary tangles (NFTs), the major pathological features in AD comprise brain accumulation of amyloid β peptide (Aβ) as senile plaques and in the form of cerebral amyloid angiopathy (CAA).^1^ The deposition of Aβ in cerebral blood vessels influences vascular function and worsens the pathology contributing to cognitive decline.^2^–^6^ Recent studies suggest reduced Aβ clearance from the brain rather than increased Aβ production is responsible for Aβ accumulation in the brain in the common late-onset form of AD.^7^ Aβ may be degraded by glial cells,^8^,^9^ and proteases (e.g., by neprilysin or insulin degrading enzyme)^10^ and is transported across the endothelium by the low-density lipoprotein receptor-related protein 1 (LRP-1) or the receptor for advanced glycation end products (RAGE).^11^,^12^ The drainage of extracellular Aβ along the basement membranes of capillaries and arteries appears as the most important mechanism for removal of Aβ from the brain.^3^,^13^,^14^ Besides impacting on the structural and functional components of the neurovascular unit, deposition of Aβ within the perivascular drainage pathway lowers the motive force of Aβ clearance, thereby contributing to increased parenchymal Aβ deposition.^15^,^16^ Indeed, a recent study using real-time imaging showed direct evidence that tracers injected into the living mouse brain were cleared along arteries and capillaries but not veins.^17^ Solute clearance along the perivascular route was impaired in mice with hemodynamic insufficiency as well as in APPswe/PS1dE9 transgenic mice, which develop parenchymal and microvascular amyloid deposits.^17^ Consistent with such findings, immunotherapy intended to reverse the accumulation of Aβ plaques in the brain has been shown to concomitantly increase cerebrovascular Aβ and exacerbate microvascular lesions.^18^,^19^ Unusually high levels of soluble Aβ have been also detected in the brains of immunized AD patients.^20^ These results suggest that Aβ immunization may result in the failure of efficient perivascular drainage of Aβ solubilized from plaques resulting in an increase in the severity of CAA.^18^,^21^ Thus, approaches that enhance Aβ clearance via perivascular drainage and protect the neurovascular unit hold promise for the development of novel therapies for AD.

Cyclic nucleotide phosphodiesterases (PDEs) play critical roles in regulating intracellular cyclic nucleotides (cyclic adenosine monophosphate (cAMP) and cyclic guanosine monophosphate), which are important second messengers involved in intracellular signal transduction in all tissues. Thus far, 11 PDE families have been described, most of them are expressed in the brain, attracting attention as a source of new targets for the treatment of psychiatric and neurodegenerative disorders.^22^,^23^ PDE III, PDE IV, or PDE V inhibitors have been shown to have positive effects on the pathology and behavior in several animal models of AD^22^–^25^ and human studies of AD patients.^26^,^27^ Most of these studies were based on the perspective of neuronal/synaptic and glial dysfunction, and impairment of neurogenesis and synaptic resilience in AD models and patients. However, considering that vascular impairment in the context of neurovascular unit is also closely associated with the pathogenesis of AD/CAA^28^,^29^ and the failure of Aβ immunotherapy,^18^,^20^ restoring vascular integrity in addition to neuronal/synaptic and glial function should be crucial for the treatment of AD/CAA.

PDE III is the major cAMP-hydrolyzing PDE (a negative regulator of cAMP) uniquely expressed in vascular smooth muscle cells, and PDE IIIA isoforms are also involved in cardiovascular function by regulating vascular smooth muscle growth regulation and phenotypic changes. Cilostazol, a selective inhibitor of PDE III, increases cAMP in vascular cells and has multiple effects on the vasculature such as vasodilatation, anti-oxidation, anti-inflammation, regulation of smooth muscle cell,^30^ increase in cerebral hemodynamics,^31^ pulse duration time^32^ and arterial elasticity^33^ with maintenance of microvascular integrity.^34^ We have recently shown that patients with mild cognitive impairment receiving cilostazol exhibit significantly reduced cognitive decline,^35^,^36^ suggesting that cilostazol, originally a drug for stroke and peripheral arterial disease, is effective for dementing disorders through its action on the vasculature and providing rationale of experimental study using an animal model of cerebrovascular β-amyloidosis.

Given the rationale and background outlined above, we first explored the expression of PDE III in postmortem human brains followed by a series of experiments using transgenic mice (Tg-SwDI mice) expressing the Swedish (K670/M671L) and vasculotropic Dutch/Iowa (E693Q/D694N) mutant of the human amyloid precursor protein (APP) to examine whether cilostazol protected against the disruption of the neurovascular unit and rescued the perivascular drainage of Aβ in AD/CAA. We further investigated whether cilostazol is beneficial or improves vascular reactivity, cerebral blood flow (CBF), perivascular Aβ drainage, vascular morphology, and behavioral/cognitive function in the transgenic mice, which exhibit deficient cerebral clearance of Aβ.^37^ Furthermore, we examined whether cilostazol can alter the Aβ biogenesis in cultured neurons as well as the in vivo system. These experimental approaches provide strong rationale for placebo-controlled pharmacological trials to establish the efficacy of cilostazol in patients with AD/CAA.

## Subjects/Materials and Methods

### Postmortem human brain material

Autopsied brains were obtained from Kyoto University Hospital through a process approved by an institutional research committee. Methods and relevant references are available in the Data S1.

### Experimental study

We used transgenic mice, C57BL/6-Tg(Thy1-APPSwDutIowa) BWevn/J (Jackson Laboratory, Bar Harbor, ME) as a CAA mouse model. Experimental details are available in the Data S1.

## Results

### PDE III, a negative regulator of cAMP, was abnormally expressed in the cerebral vessels of AD/CAA patients and correlated with the vascular amyloid burden

**Figure 1:**
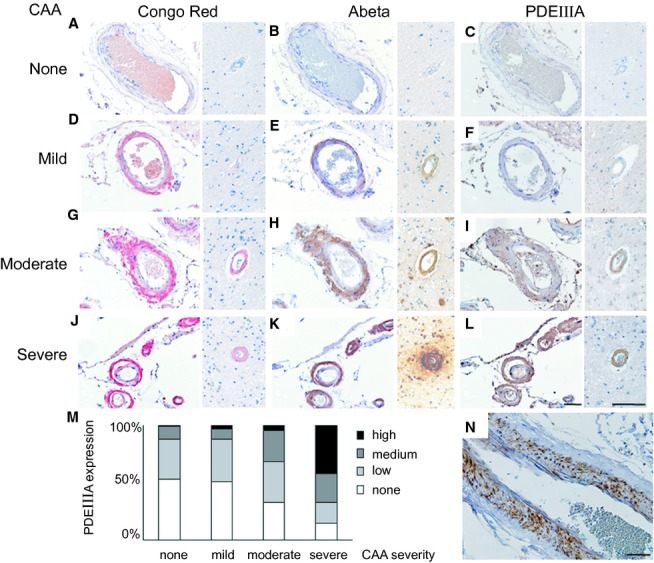
PDE IIIA expression is upregulated in the vascular wall in close correlation with CAA severity. (A–L and N) Representative images of Congo red staining (A, D, G and J), Aβ immunostaining (B, E, H and K), and PDE IIIA immunostaining (C, F, I, L and N) in the leptomeningeal (left) and intracerebral cortical (right) vessels of patients without CAA (A–C) and with mild CAA (D–F), moderate CAA (G–I), and severe CAA (J–L and N). Scale bar indicates 100 μm. (M) Histogram showing the percentage of vessels with PDE IIIA expression (none, low, medium, and high) in patients without CAA and with mild, moderate, and severe CAA. PDE, phosphodiesterases; CAA, cerebral amyloid angiopathy.

On the basis that PDE III is a vasoactive enzyme uniquely expressed in vascular smooth muscle cells and plays a pivotal role in vascular function, we evaluated PDE IIIA immunoreactivity in postmortem human brains. The demographic details and pathological findings are summarized in Table S1. PDE IIIA immunostaining was negligible in the brain without CAA (Fig. 1A–C). In contrast, PDE IIIA-immunoreactivity was upregulated mostly in the vascular smooth muscle cells, of both the leptomeningeal and cortical arteries as CAA severity increased (Fig. 1D–L and N) with a significant linear correlation (*Rs* = 0.8272, *P* < 0.001 for Spearman's rank correlation coefficient, Fig. S1). Two relatively pure CAA cases (Case No. 9 and 15; moderate and severe, respectively) showed increased PDEIII expression, while two controls (Case No. 16 and 17) without brain pathology showed lower PDEIII expression score (Table S1). PDE IIIA was also detected in the parenchyma (Fig. S2A–C). Some of the cortical capillaries exhibited PDE IIIA immunostaining in the vascular walls suggestive of pericytes (Fig. S2D and E). Some of neurons including dystrophic neurites around the amyloid core showed PDE IIIA expression (Fig. S3E–L). However, neuropil threads and amyloid cores did not express PDE IIIA (Fig. S3E–L). The aged Tg-SwDI mice also showed the increased expression of PDE IIIA in the vascular wall of the leptomeningeal and intracortical arteries (Fig. S4). These results suggested that PDE IIIA expression is abnormally upregulated especially in the vascular smooth muscle cells in close correlation with the vascular amyloid burden.

### Cilostazol restored vasoreactivity in Tg-SwDI mice

**Figure 2:**
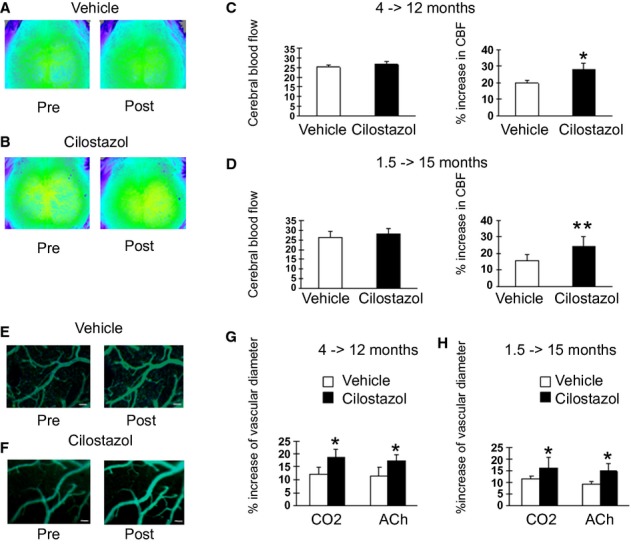
Cilostazol-treated Tg-SwDI mice exhibit increased cerebral blood flow response to hypercapnia and increased vasodilative response to hyperacapnia and acetylcholine. (A and B) Representative images showing temporal changes of cerebral blood flow (CBF) before (Pre) and after (Post) hypercapnia in vehicle-treated and cilostazol-treated Tg-SwDI mice. (C) Histogram showing CBF (left) and % increase in CBF (right) in vehicle-treated (*n* = 3) and cilostazol-treated (*n* = 3) Tg-SwDI mice aged 12 months treated from aged 4 months. (D) Histogram showing CBF (left) and % increase in CBF (right) in vehicle-treated (*n* = 7) and cilostazol-treated (*n* = 9) Tg-SwDI mice aged 15 months treated from aged 1.5 months. Error bars indicate SD. **P < 0.01 and *P < 0.05 in vehicle-treated Tg-SwDI mice versus cilostazol-treated Tg-SwDI mice. (E and F) Representative images showing temporal changes of leptomeningeal arteries before (left) and after (right) hypercapnia in vehicle-treated (E) and cilostazol-treated (F) mice. Scale bars indicate 50 μm. (G) Histogram showing % increase of vascular diameter in response to hypercapnia (left) and acetylcholine (ACh, right) in vehicle-treated (*n* = 3) and cilostazol-treated (*n* = 3) Tg-SwDI mice aged 12 months treated from aged 4 months. (H) Histograms showing % increase of vascular diameter in response to hypercapnia (left) and acetylcholine (right) in vehicle-treated (*n* = 4) and cilostazol-treated (*n* = 5) Tg-SwDI mice aged 15 months treated from 1.5 months. Error bars indicate SD. *P < 0.05 in vehicle-treated Tg-SwDI mice versus cilostazol-treated Tg-SwDI mice.

Impairments in cerebral circulation and vascular reactivity have substantial roles in the onset and progression of AD/CAA in human^38^ and mouse models of AD/CAA including Tg-SwDI mice.^5^,^39^ Consistent with the previous reports, which demonstrated that the Tg-SwDI mice showed microvascular Aβ deposition throughout the forebrain by 12 months of age,^37^ most of the vessels were affected by microvascular amyloid in vehicle-treated Tg-SwDI mice aged 23 months (Fig. S5). We first investigated whether cilostazol ameliorates vascular dysfunction in Tg-SwDI mice. Resting CBF was marginally increased in cilostazol-treated Tg-SwDI mice compared with vehicle-treated Tg-SwDI mice both aged 12 months (8-month cilostazol treatment vs. vehicle; 26.7 ± 1.3 vs. 25.1 ± 1.1, *P* = 0.13) and aged 15 months (13.5-month cilostazol treatment on vs. vehicle; 27.9 ± 2.9 vs. 26.1 ± 3.2, *P* = 0.09) (Fig. 2A–D; left panels) but these increments did not reach statistical significance. However, cilostazol-treated mice showed a significant increase in CBF response to hypercapnia compared with vehicle-treated Tg-SwDI mice both aged 12 months (8-month cilostazol treatment vs. vehicle; 28.0 ± 4.3% vs. 19.6 ± 2.2%; *P* = 0.0495) and aged 15 months (13.5-month cilostazol treatment vs. vehicle; 24.3 ± 6.1% vs. 15.5 ± 3.9%, *P* < 0.01) (Fig. 2A–D; right panels). We evaluated the vasodilatory response to hypercapnia and acetylcholine using in vivo imaging (Fig. S6). We assessed the vessels that are comparable in the baseline vascular diameters between the treated and untreated groups (Fig. 2E and F; left panels). However, cilostazol-treated Tg-SwDI mice showed significant increases in the vasodilatory response to hypercapnia (Fig. 2E and F; right panels) and acetylcholine both aged 12 months (8-month cilostazol treatment vs. vehicle; 19.0 ± 3.2% vs. 12.1 ± 2.9% for hypercapnia, *P* = 0.0495; 17.4 ± 2.3% vs. 11.2 ± 3.6%, *P* = 0.0495 for acetylcholine) (Fig. 2G) and aged 15 months (13.5-month cilostazol treatment vs. vehicle; 16.3 ± 4.5% vs. 11.5 ± 1.1% for hypercapnia, *P* < 0.01; 14.7 ± 3.2% vs. 9.3 ± 0.8% for acetylcholine, *P* = 0.032) (Fig. 2H). This was consistent with the results of CBF response. These findings indicated that cilostazol restores cerebral hemodynamic reserve in Tg-SwDI mice.

### Cilostazol facilitated perivascular drainage of Aβ in Tg-SwDI mice

**Figure 3:**
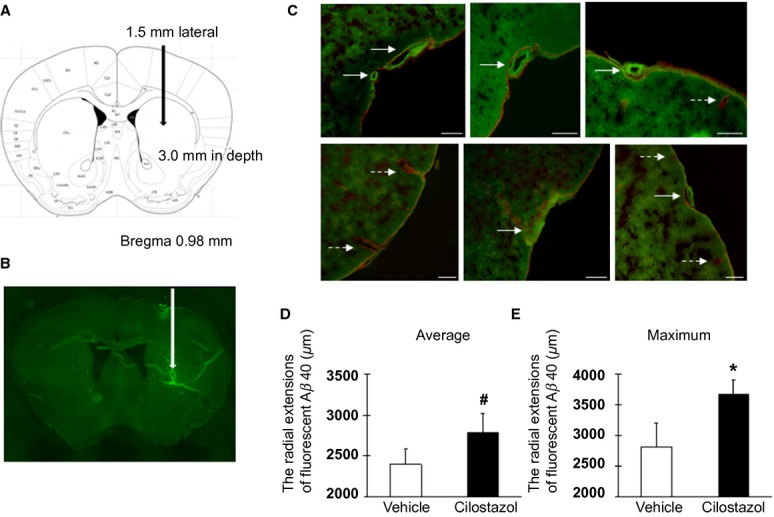
Facilitation of perivascular drainage of Aβ in cilostazol-treated Tg-SwDI mice. (A and B) A scheme (A) and coronal image (B) showing the site of the soluble fluorescent Aβ_1-40_ injection (striatum; 0.98 mm anterior and 1.5 mm lateral from bregma, 3.0 mm in depth from the dorsal surface of the brain). (C) Representative double immunofluorescence images for laminin (red)/fluorescent Aβ_1-40_ (green) in the leptomeningeal vessels 30 min after the injection of fluorescent Aβ_1-40_ into the striatum. Solid arrow and dashed arrow indicate Aβ_1-40_-positive and Aβ_1-40_-negative staining, respectively. Scale bars indicate 100 μm. (D and E) Histograms showing the averaged (D) and maximal (E) values of radial extensions of fluorescent Aβ_1-40_ in the leptomeningeal vessels from the site of injection (*n* = 3–4 for each group). Error bars indicate SD. *P < 0.05 and #*P* < 0.1 in vehicle-treated Tg-SwDI mice versus cilostazol-treated Tg-SwDI mice.

Cerebrovascular dysfunction and reduced arterial pulsations may result in the failure of perivascular drainage of soluble Aβ, leading to the accumulation and aggregation of Aβ in the vessel walls and in the brain parenchyma.^17^ We investigated whether the restoration of vascular reactivity by cilostazol led to an improvement of perivascular drainage of soluble Aβ in Tg-SwDI mice. Intracerebral injections of soluble fluorescent HiLyte Aβ tracers were performed, following the methodology described before^13^ (Fig. 3A). Thirty minutes after the injection into the striatum, soluble fluorescent Aβ_1-40_ had spread diffusely throughout the brain parenchyma and was present within the basement membranes of parenchymal and leptomeningeal arteries. At 30 min post-injection, most of the Aβ had drained out of the brain from the site of injection and Aβ was detected only in the leptomeningeal arteries (Fig. 3B), and within Iba-1-positive microglia as well as NeuN-positive neurons (data not shown). Within the leptomeningeal arteries, Aβ was located outside the platelet endothelial cell adhesion molecule-1-positive endothelial cells and colocalized with smooth muscle actin-positive vascular smooth muscle cells (data not shown) and the laminin present in the basement membranes surrounding smooth muscle cells (Fig. 3C). The radial spread of Aβ localized in the leptomeningeal arteries from the injection site was calculated. We did not quantify the fluorescent Aβ -positive vessels around the needle tract as they could be technical artifacts. The number of vessels analyzed was similar for vehicle- and cilostazol-treated Tg-SwDI mice (cilostazol vs. vehicle; 31 ± 3.7 vs. 29 ± 7.0). There were marginally significant and significant differences in the averaged and maximal radial spread of fluorescent Aβ_1-40_, respectively, between vehicle- and cilostazol-treated Tg-SwDI mice at 15 months of age (13.5-month cilostazol treatment vs. vehicle; 2783 ± 234 μm vs. 2395 ± 186 μm for average spread, *P* = 0.057; 3678 ± 238 μm vs. 2820 ± 386 μm for maximum spread, *P* = 0.028) (Fig. 3D and E). These results suggest that long-term cilostazol treatment helps to maintain efficient perivascular drainage of Aβ in Tg-SwDI mice.

### Cilostazol reduced Aβ deposits in the Tg-SwDI mice brain

**Figure 4:**
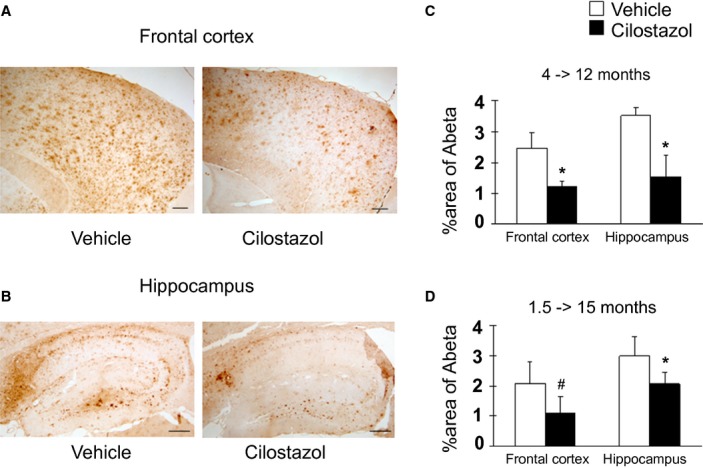
Cilostazol-treated Tg-SwDI mice exhibit reduced levels of Aβ deposits compared with vehicle-treated Tg-SwDI mice. (A and B) Representative images of Aβ staining in the frontal cortex (A) and hippocampus (B) in Tg-SwDI mice treated with vehicle (left) or cilostazol (right). Scale bars indicate 400 μm in A and 200 μm in B. (C and D) Histogram showing the density of cells immunoreactive for Aβ in the frontal cortex (left) and hippocampus (right) of vehicle-treated and cilostazol-treated Tg-SwDI mice aged 12 months treated from 4 months (C) and aged 15 months treated from 1.5 months (D) (*n* = 3–5 for each group). Values are expressed as mean ± SD. *P < 0.05 and #*P* < 0.1 in vehicle-treated Tg-SwDI mice versus cilostazol-treated Tg-SwDI mice.

Tg-SwDI mice develop robust accumulation of both vascular and parenchymal Aβ deposits with predominance of Aβ_1-40_ over Aβ_1-42_ in the perivascular/vascular areas starting at 3 to 5 months of age (Fig. S7A–D).^37^ We hypothesized that facilitation of perivascular Aβ drainage with cilostazol reduces Aβ deposits. Thus, we assessed the effects of cilostazol on Aβ accumulation in the Tg-SwDI mice brain. Cilostazol-treated Tg-SwDI mice showed significantly or marginally significantly decreased immunoreactivity of Aβ in the frontal cortex (Fig. 4A) and hippocampus (Fig. 4B) compared with vehicle-treated Tg-SwDI mice both aged 12 months (frontal cortex, *P* = 0.0495; hippocampus, *P* = 0.0495) (Fig. 4C) and 15 months (frontal cortex, *P* = 0.057; hippocampus, *P* = 0.029) (Fig. 4D). Both vehicle- and cilostazol-treated Tg-SwDI mice did not show apparent microhemorrhage aged 15 months (Fig. S7E and F). The evaluation of the blood brain barrier (BBB) integrity using Evans blue also showed that the Tg-SwDI mouse aged 10 months did not exhibit apparent Evans blue extravasation into the central nervous system (CNS) parenchyma; however, Evans blue did diffuse throughout the extracellular space in non-neural tissue (e.g., kidney, heart, and liver) (Fig. S8). Taken together, the above results indicated that cilostazol reduces Aβ accumulation in the Tg-SwDI mice brain possibly by promoting perivascular drainage of Aβ associated with preserved hemodynamic reserve.

### Cilostazol attenuated the degradation of vascular walls with Aβ deposit

**Figure 5:**
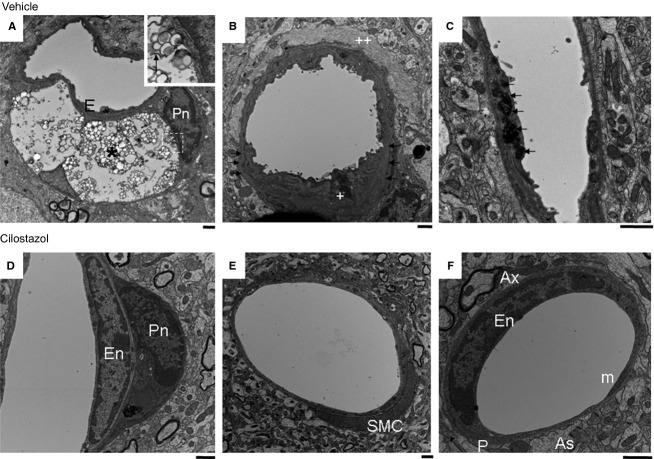
Electron microscopic images of vehicle-treated (A–C) and cilostazol-treated (D–F) Tg-SwDI mouse aged 21 months. The vehicle-treated Tg-SwDI mice showed marked degenerative changes of cerebral vessels, which were suppressed by long-term cilostazol treatment. (A) Numerous large vesicles and empty space were present in the cytoplasm of pericytes (*). *Inset*: Higher magnification of multiple vesicles (dashed square). The arrow indicates isolation membrane. (B) Degenerative changes of smooth muscle cells (+) accompanied with thickening/alterations of basement membrane (arrows) and amyloid fibril deposit (++). (C) Endothelial degeneration indicated by damaged intracellular organelles, replaced by dark materials (arrows). (D) Mild degenerative changes of pericyte were seen. Small vacuoles and dark materials in the cytoplasm of pericyte were present. (E) Almost intact arteriole. (F) Almost intact capillary. Scale bars indicate 1 μm. As, astrocyte; Ax, axon; BM, basement membrane; E, endothelial cell; En, nucleus of endothelial cell; m, mitochondrion; P, pericyte; Pn, nucleus of pericyte; SMC, smooth muscle cell.

To examine the nature of Aβ accumulation and morphological changes in detail, we performed electron microscopy of Tg-SwDI mice aged 21 months that had been treated with cilostazol or vehicle for 17 months. Whereas 25% (49 per 195) of the vessels in the vehicle-treated Tg-SwDI mice showed some type of abnormality in the vascular wall vis-a-vis degeneration of pericytes or vascular smooth muscle cells or damaged intracellular organelles and alterations in the basement membranes (Fig. 5A–C, and Fig. S9A–D), only 6.4% (10 vessels per 155) of the vessels was affected in the cilostazol-treated mice (Fig. 5D–F, and Fig. S9E and F). The majority of degenerating pericytes in vehicle-treated Tg-SwDI mice exhibited morphological changes related to severe cell injury. These were apparent as numerous large vesicles, membranes around portions of the cytoplasm (called isolation membrane), mitochondrial injury, lysosomal inclusions, and large lysosomes were found in the cytoplasm of pericytes, mostly markers of autophagy (Fig. 5A, and Fig. S9A and B). The arterioles of vehicle-treated Tg-SwDI mice showed a similar degeneration of vascular smooth muscle cells with vacuolization and destroyed organelles accompanied by basement membrane thickening/alterations (Fig. 5B, C, and Fig. S9C, D). Conversely, cilostazol-treated Tg-SwDI mice only rarely showed the above-abnormal findings (Fig. 5D–F, and Fig. S9E and F).

### Cilostazol prevented the decline of cognitive and grooming performance in Tg-SwDI mice

**Figure 6:**
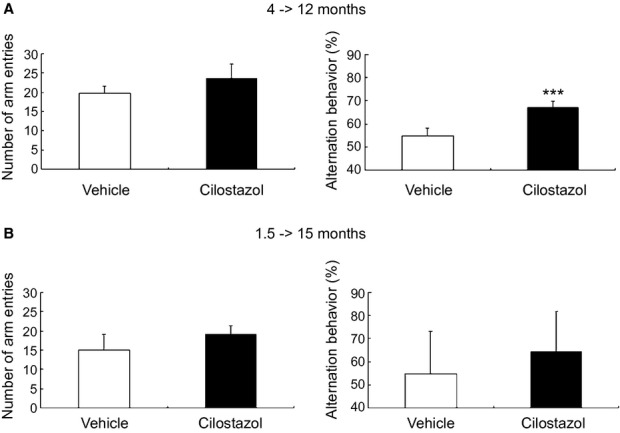
Cilostazol prevents working memory deficits in Tg-SwDI mice. (A) Histograms showing a number of arm entries (left) and alternation behavior (right) in the Y maze test of vehicle-treated (*n* = 11) and cilostazol-treated (*n* = 11) Tg-SwDI mice aged 12 months treated from 4 months. (B) Histograms showing number of arm entries (left) and alternation behavior (right) in the Y maze test of vehicle-treated (*n* = 10) and cilostazol-treated (*n* = 10) Tg-SwDI mice aged 15 months treated from 1.5 months. Error bars indicate SD. ***P < 0.001 in vehicle-treated Tg-SwDI mice versus cilostazol-treated Tg-SwDI mice.

To evaluate cognitive function, we examined behavioral performance by using the Y maze test. Alternations of entries in the arms of the Y maze were significantly increased in cilostazol-treated Tg-SwDI mice (67.0 ± 2.9%) compared with vehicle-treated mice (54.9 ± 3.3%) aged 12 months (*P* < 0.001; Fig. 6A, right panel). Spontaneous activity was not significantly different between the two groups of mice (Fig. 6A, left panel). At 15 months of age, although there was a trend for increase in alternations of entries in cilostazol-treated mice, no significant difference in both alternations of entries in the arms and spontaneous activity between vehicle- and cilostazol-treated Tg-SwDI mice (Fig. 6B). There were no significant differences in the number of total entries and alternations of entries between vehicle- and cilostazol-treated wild type mice aged 12 months (Fig. S10). Cilostazol-treated Tg-SwDI mice showed significantly better hair condition compared with vehicle-treated Tg-SwDI mice aged 15 months, also suggesting that cilostazol prevents cognitive decline in Tg-SwDI mice (Data S1 and Fig. S11).

### Cilostazol suppressed endogenous Aβ production in the primary cultured neurons

**Figure 7:**
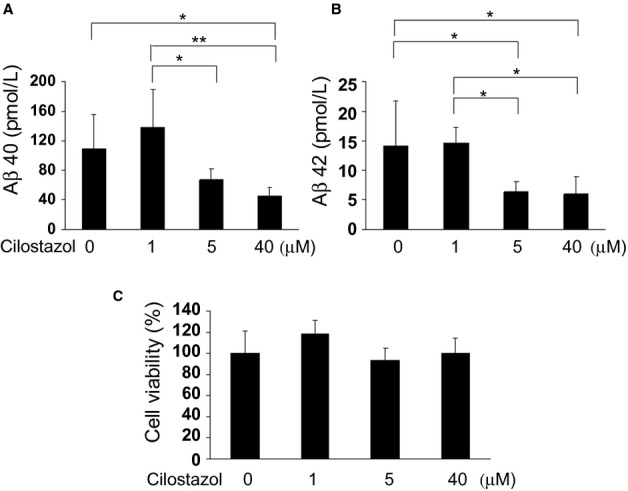
Cilostazol suppresses endogenous Aβ40 and Aβ42 production from cultured neurons. (A and B) The concentrations of Aβ40 (A) and Aβ42 (B) in the conditioned media from cultured rat neurons were measured by ELISA at 48 h after treatment with different concentration of cilostazol (1–40 μM). (C) WST assay using samples from cultured rat neurons at 48 h after treatment with different concentration of cilostazol (1–40 μM). *N* = 6 each. Values are mean ± SD. *P < 0.05, * *P < 0.01.

There is a possibility that the observed reduction in Aβ deposition is a consequence of decreased Aβ production, in addition to increased Aβ clearance. Indeed, a previous study showed that cilostazol decreased extracellular and intracellular Aβ levels in mouse neuroblastoma N2a cells expressing human APP with Swedish mutation.^25^ We examined the effect of cilostazol on endogenous Aβ production from neurons in vitro using the primary cultured rat neurons. This showed that cilostazol decreased the amount of Aβ40 and Aβ42 in the media released from neurons in a dose-dependent manner (Fig. 7A and B). A WST assay showed no significant differences among these groups (Fig. 7C). These results suggest that cilostazol can suppress the endogenous Aβ production from neurons without affecting cell viability.

### Cilostazol did not affect APP processing in Tg-SwDI mice

**Figure 8:**
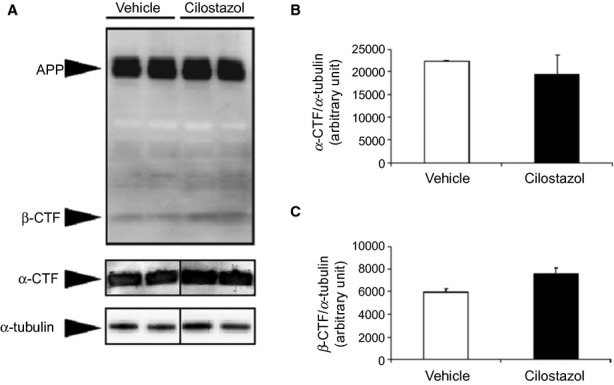
Cilostazol does not alter amyloid precursor protein (APP) processing in Tg-SwDI mice. (A) Western blot images of full length APP, APP C-terminal fragments (CTFs), β-CTF and α-CTF, and α-tubulin using protein extracts from the brain tissue of vehicle- and cilostazol-treated Tg-SwDI mice (*n* = 2–3 for each group) aged 15 months receiving vehicle or cilostazol for 13.5 months. For full-length APP, β-CTF, and α-tubulin, 50 μg protein lysates were applied in each well while 5 μg of protein lysates were applied to circumvent “white bands” for α-CTF. (B and C) Semiquantitative analysis of α-CTF (B) and β-CTF (C) normalized for α-tubulin. Values are mean ± SD.

Although cilostazol does reduce neuronal Aβ production, it has been established that only a very minor fraction of cilostazol can cross BBB and enter the brain after the peroral administration.^40^ Therefore, the direct effects of cilostazol on neurons were expected to be less significant in vivo than in vitro. Based on the knowledge that β-secretase activity is augmented by hypoperfusion,^41^ which may be ameliorated with cilostazol administration, we performed quantitative immunoblotting using antibody against APP C-terminal domain, as a marker of APP processing. There were no significant differences in the levels of APP and APP C-terminal fragments (CTFs) between vehicle- and cilostazol-treated Tg-SwDI mice aged 15 months, which had received vehicle or cilostazol for 13.5 months (Fig. 8).

## Discussion

The complex expression patterns and activity of the different PDEs throughout the CNS under normal and pathological conditions have been the subject of intense interest during the past decade, especially in regard to the therapeutic potential of PDE inhibitors for CNS disorders.^22^,^23^ We found that PDE IIIA expression in the microvessels, predominantly in the smooth muscle cell layers, was markedly upregulated as CAA severity increased. To our knowledge, this is a first report showing the upregulation of PDE IIIA expression in the microvessels in close correlation with vascular amyloid burden. Further studies, including in vitro studies, are needed to clarify whether the increased PDE IIIA expression is a cause or consequence of amyloid deposit.

Our experimental study demonstrated that cilostazol, PDE III inhibitor, restores vascular reactivity and hemodynamic reserve, promotes perivascular drainage of Aβ, reduces degenerative changes of vascular walls with Aβ deposits and prevents the decline of cognitive performance in the Tg-SwDI mice, a model for cerebrovascular β-amyloidosis. These protective roles of cilostazol against Aβ-induced neurodegeneration seem to be mediated by its vasculotropic, but not antiplatelet, effects, as long-term aspirin treatment did not reverse vascular Aβ deposition, hemodynamic derangements, or cognitive decline in Tg-SwDI mice (unpublished data). Thus, besides its antiplatelet actions, the maintenance of neurovascular integrity with cilostazol could represent a promising approach in the decelerating cognitive decline associated with Aβ deposition. Since the motive force for perivascular Aβ drainage appears to be generated by arterial pulsations,^42^,^43^ the direct action of cilostazol on the vascular smooth muscle cells to increase pulse duration time^32^ and arterial elasticity^33^ may have contributed to the facilitated perivascular drainage of Aβ and the above positive effects of cilostazol in the Tg-SwDI mice. Furthermore, as electron microscopic examinations have revealed, the protection of pericytes and other vascular components by cilostazol may have led to the preservation of vasodilative and hemodynamic reactivity and BBB integrity along the perivascular drainage pathway.

The CNS is devoid of conventional lymphatic vessels, unlike other organs that contain networks of lymphatic vessels, which process various elements such as wastes, fluid, proteins, and cells from tissues to lymph nodes.^15^,^42^ However, the perivascular drainage system in the brain performs the main function assigned to systemic lymphatic vessels.^13^–^15^,^42^,^44^ Animal studies using various tracers have demonstrated that interstitial fluid and solutes drain rapidly via perivascular “lymphatic” pathways from brain parenchyma along basement membranes in the walls of capillaries and arteries in the opposite direction of the arterial blood flow to cervical lymph nodes.^15^,^17^,^42^ This drainage route corresponds very closely with the distribution of Aβ in the basement membranes of capillary and artery walls in CAA.^45^ The failure of this drainage in the ageing brain and in the presence of CAA results in the accumulation of insoluble and soluble Aβ and probably other metabolites that would lead to loss of homeostasis of the neuronal environment.^15^,^42^ This notion is also supported by the experimental data that the vascular Aβ deposition is increased following bilateral common carotid artery stenosis of CAA model mice^46^ or middle cerebral artery occlusion model mice.^47^ Such “lymphatic” congestion of the brain may be improved by vasoactive cilostazol.

Nevertheless, there is a possibility that the observed reduction in Aβ deposition could largely be a consequence of decreased Aβ production, rather than increased Aβ clearance. According to previous studies, cilostazol could regulate APP processing via the cAMP-PKA pathway or activation of CREB cascade,^26^ and PKA has been implicated in the regulation of APP processing.^48^ In addition, the PDE V inhibitor sildenafil has been shown to reduce brain Aβ through the cGMP-CREB pathway.^49^ Given that the activation of the 5-HT4 receptor could regulate α-secretase activity, which is associated with cAMP functional response,^50^ cilostazol-mediated cAMP might enhance α-secretase activity, leading to decreased Aβ production. Indeed, the current study showed that cilostazol could decrease endogenous Aβ generation from the cultured neurons in vitro. There is also a possibility that the improvement of cerebral hemodynamics by cilostazol could reduce in Aβ synthesis as hypoxia/hypoperfusion has been shown to upregulate β-secretase BACE1.^41^ However, cilostazol did not alter the levels of APP CTFs in Tg-SwDI mice in vivo. This discrepancy could be attributed to the poor ability of cilostazol to cross the BBB,^40^ as well as the finding that BBB was not apparently disrupted in middle-aged Tg-SwDI mice in the current study. It is nonetheless possible that cilostazol, even at a low level, activates cAMP-PKA signaling with a similar mechanism to enriched environment, leading to the activation of β2-adrenergic receptors and inhibition of synaptotoxicity of human Aβ oligomers.^51^ Taken together, the positive effects of cilostazol in vivo observed in the current study may predominantly result from facilitated Aβ clearance due to the sustained cerebrovascular function; however, the previously unidentified mechanisms leading to Aβ reduction, could offer a potential source of future investigation.

The use of Tg-SwDI mice has two distinctive advantages, when compared to other types of Tg mice harboring mutant APP. Firstly, Tg-SwDI mice have been shown to express mutant human APP at low levels, even below those of endogenous mouse APP in the brain. Despite the low expression levels of mutant APP, Tg-SwDI mice develop early-onset robust accumulation of Aβ in the brain. This could result from the deficient clearance of Dutch/Iowa mutant Aβ from the CNS at the cerebral vasculature, as shown in the previous studies.^37^,^52^ Since decreased elimination, rather than increased production, of Aβ is likely to be a major cause of sporadic late-onset AD, this model would be useful for investigating the mechanisms and therapeutic approaches for sporadic AD. Second, Dutch/Iowa mutant Aβ peptides have highly vasculotropic nature and Tg-SwDI mice exhibit extensive microvascular amyloid deposition. A recent report showed that Tg-SwDI mice, which dominantly develop microvascular amyloid exhibit cognitive dysfunction at 3 months of age. However, Tg-5 × FAD mice that dominantly develop parenchymal amyloid deposits did not exhibit cognitive dysfunction at this age. These findings suggest that cerebral microvascular pathology might contribute to the early stages of cognitive impairment in AD and related disorders,^53^ consistent with the notion that cerebral vascular dysfunction is an early contributor for AD pathophysiology.^2^ The positive effects of cilostazol on Tg-SwDI mice in the current study suggest that cilostazol is a promising therapeutic target for early stages of cognitive impairment of neurodegenative etiology.

There are several issues to be addressed in future studies. First, we focused on only middle-aged and old Tg-SwDI mice, although emerging evidence shows that early vascular changes may precede Aβ accumulation and progressive disease cascades. Further studies are needed to elucidate whether cilostazol can improve early vascular alterations in young Tg-SwDI mice. Second, we specifically focused on perivascular drainage of Aβ. However, other clearance mechanisms, such as transcytotic delivery across BBB or degradation of Aβ by microglial/astroglial cells or enzymes, might contribute to reduction of Aβ overload. In addition, clearance pathways other than perivascular lymphatic routes, such as paravenous routes and bulk fluid flow through astrocytic endfeet^54^ or paraaxonal/neuronal pathways, should be considered in future studies. Finally, the current study shows that cilostazol reduces Aβ overload by hemodynamic enhancement. However, the accumulation of NFTs within neurons, composed largely of ubiquitin and the microtubule-associated protein tau, is another important hallmark of AD.^1^ While the accumulation of Aβ may derive mainly from the failure of the clearance system as mentioned above, that of NFTs appears to be associated at least partially with the failure of the ubiquitin-proteasome system.^16^ Additionally, hemodynamic insufficiency has been shown to enhance phosphorylation of tau through upregulation of tau phosphorylating enzymes.^55^ Further studies are warranted to examine whether cilostazol have positive effects on the neuropathology of NFTs as well as Aβ.

In conclusion, our study showed that the vasoactive drug cilostazol, a PDE III inhibitor, prevented cognitive decline triggered with Aβ deposition by facilitating Aβ clearance out of the brain and preserving neurovascular unit. Cilostazol, which can resolve the failure of the Aβ drainage pathway may provide a novel promising therapeutic target for AD/CAA, potentially in combination with early Aβ immunization therapy and pharmacological intervention to enhance enzymatic degradation of Aβ and absorption of Aβ into the blood. A prospective trial is now needed to determine the effects of cilostazol on AD/CAA.

## Supporting Information

**Data S1.** Supplemental materials. **Data S1.** Supplemental data. **Figure S1.** The correlation between the CAA severity and the PDE IIIA expression score demonstrates significant linear correlation (*Rs* = 0.8272, *P* < 0.001). PDE IIIA expression score was calculated from the average of qualified grading of PDE IIIA expression (0 = none, 1 = low, 2 = medium, 3 = high). **Figure S2.** (A–E) Representative images of PDE IIIA immunostaining in patients with AD and CAA. PDE IIIA expression is only slightly observed in neurites in the cerebral cortex (A) and pyramidal neurons in the hippocampal CA3 regions (B) and dentate gyrus (C), whereas it is strong in the cortical vessels in patients with AD/CAA (D, E). Scale bar indicates 50 μm. **Figure S3.** Representative images of the leptomeningeal arteries (A–D), the cerebral cortex (E–H), and the senile plaques (I–L), which were stained for Aβ (A, E, I), paired helical filament-tau (PHF-tau) (B, F, J), PDE IIIA (C, G, K), and Congo red (D, H, L) in patients with AD and CAA. Of note, some neurons with dystrophic neurites (J, dashed arrow) showed PDE IIIA expression (G, K), but neuropil threads (F, arrows) and amyloid cores (I and L, arrows) were not positive for PDE IIIA. Scale bar indicates 50 μm. **Figure S4.** PDE IIIA expression is increased in the vascular wall in aged Tg-SwDI mouse. Representative images of PDE IIIA immnostaining in the leptomeningeal (A–C) and intracortical (D–F) arteries of wild type mouse aged 12 months (A and D), Tg-SwDI mouse aged 4 months (B, E), and Tg-SwDI mouse aged 23 months (C and F). Scale bar indicates 20 μm. **Figure S5.** Thioflavin-S staining in the Tg-SwDI mouse aged 23 months. Scale bars indicate 100 μm in A–F, 1 mm in G, and 500 μm in H and I. **Figure S6.** Representative images of vehicle- (A) and cilostazol-treated (B) Tg-SwDI mice showing where vessel diameter was quantified. An averaged vessel diameter across a 25 μm longitudinal segment (8 consecutive segments per mouse) was analyzed before (baseline) and after inhalation of 5% CO_2_ or treatment with acetylcholine. Scale bars indicate 50 μm. **Figure S7.** Tg-SwDI mice exhibit robust accumulation of both vascular and parenchymal Aβ with predominance of Aβ40 over Aβ42 in the perivascular/vascular areas without apparent microhemorrhage or BBB leakage at 15 months of age. (A–D) Representative images of Aβ40 (A), Aβ42 (B), and Aβ (6E10) immunostaining (C and D), Perls-Stieda's iron staining (E), and IgG staining (F) in Tg-SwDI mice aged 15 months. Scale bars indicate 50 μm in A and B, 20 μm in C and D, and 100 μm in E and F. **Figure S8.** Representative macroscopic (A and B) and microscopic fluorescent (C and D) images of the brain (A, C) and kidney (B and D) in the Tg-SwDI mouse aged 10 months after Evans blue, followed by FITC dextran (green), injection. There were clear differences in the extravascular extravasation of Evans blue (shown in blue in A, B, and red in C, D) between the brain and kidney. Scale bars indicate 50 μm. **Figure S9.** Electron microscopic images of vehicle-treated (A–D) and cilostazol-treated (E, F) Tg-SwDI mice aged 21 months. (A) Multiple vesicles in the cytoplasm of pericytes. (B) Multiple vesicles (+) and damaged intracellular organelles including mitochondria replaced by dark material (upper inset) in the pericytic cytoplasm. Multiple vacuoles (++) and thickening/alterations of basement membrane (lower inset). The regions marked by dashed squares are enlarged in the right upper and lower inset, respectively. (C) Thickening/alterations of basement membrane. The region marked by a dashed square is enlarged in the inset. (D) Two transversely sectioned microvessels were seen connected by a dysmorphic process. Multiple stages of autosomes, lysosomes, and damaged intracellular organelles with disruption of mitochondrial membrane and reduced cristae (arrows, right lower inset) were present (++). The regions marked by dashed squares are enlarged in the right upper and lower inset, respectively. (E and F) Almost intact capillaries were seen in cilostazol-treated Tg-SwDI mice. Arrows indicate tight junction of endothelial cells. Pericytic mitochondria were normal. The region marked by a dashed square is enlarged (F, inset). Scale bars indicate 1 μm. As, astrocyte; Ax, axon; BM, basement membrane; E, endothelial cell; En, nucleus of endothelial cell; Ly, lysosome; m, mitochondrion; P, pericyte; Pn, nucleus of pericyte. **Figure S10.** (A) Histograms showing number of arm entries (left) and alternation behavior (right) in the Y maze test of vehicle-treated (*n* = 10) and cilostazol-treated (*n* = 10) wild type mice aged 12 months treated from 11 months of age. Error bars indicate SD. **Figure S11.** Grooming performance in vehicle-treated and cilostazol-treated Tg-SwDI mice. (A) Images showing the hair (ruffled coat) conditions graded as four categories 0–3. (B) Histogram showing grading score of the hair (ruffled coat) condition of vehicle-treated (*n* = 10) and cilostazol-treated (*n* = 12) Tg-SwDI mice aged 15 months. Error bars indicate SEM. **P* < 0.05 in vehicle-treated Tg-SwDI mice versus cilostazol-treated Tg-SwDI mice. **Table S1.** Detailed neuropathological features and analyses of 17 patients with AD or non-AD. These patients were also divided into 4 groups according to the Vonsattel grade of CAA (4 patients without CAA, 4 patients each with mild or moderate, and 5 patients with severe CAA). The grading score of PDE IIIA expression is calculated from the average of qualified grade (0 = none, 1 = low, 2 = medium, 3 = high). In total, 1700 leptomeningeal and cortical arteries (100 per patient) were analyzed in the randomly selected 5 regions (20 vessels per one region) from the temporal and occipital lobes. AD, Alzheimer's disease; ALS, amyotrophic lateral sclerosis; CAA, cerebral amyloid angiopathy; CERAD, Consortium to Establish a Registry for Alzheimer's Disease; CHF, congestive heart failure; CLL, Chronic lymphocytic leukemia; NFT, neurofibrillary tangle; SIVD, subcortical ischemic vascular dementia; VaD, vascular dementia; ND, not determined.
