# Supplementary material for: Phosphodiesterase III inhibitor promotes drainage of cerebrovascular β-amyloid
Source: Ann Clin Transl Neurol. 2014 Jul 8;1(8):519–33. doi: 10.1002/acn3.79 (PMC4184555; doi:10.1002/acn3.79)
Supplement: Supplementary file 1 [file acn30001-0519-SD1.doc]

**Supplemental materials**

**Phosphodiesterase III inhibitor promotes drainage of cerebrovascular β-amyloid**

Takakuni Maki, MD, PhD^1,2^*, Yoko Okamoto, MD, PhD^1,3^, Roxana O. Carare, MD, PhD^4^, Yoshiki Hase, MD, PhD^1^, Yorito Hattori, MD^1^, Cheryl A. Hawkes, PhD^4^, Satoshi Saito, MD^1^, Yumi Yamamoto, PhD^5^, Yasukazu Terasaki, MD, PhD^2^, Hatsue Ishibashi-Ueda, MD, PhD^3^, Akihiko Taguchi, MD, PhD^6^, Ryosuke Takahashi, MD, PhD^1^, Taihei Miyakawa, MD, PhD^7^, Raj N. Kalaria, PhD, FRCP^8^, Eng H. Lo, PhD^2^, Ken Arai, PhD^2^, Masafumi Ihara, MD, PhD, FACP^1,9^*

^1^ Department of Neurology, Graduate School of Medicine, Kyoto University, Kyoto, Japan.

^2^ Departments of Radiology and Neurology, Massachusetts General Hospital and Harvard Medical School, Charlestown, Massachusetts, USA.

^3^ Department of Pathology, National Cerebral and Cardiovascular Center, Osaka, Japan.

^4^ Division of Clinical Neurosciences, Southampton General Hospital, Southampton University, Hampshire, UK.

^5^ Department of Regenerative Medicine and Tissue Engineering, National Cerebral and Cardiovascular Center, Osaka, Japan.

^6^ Department of Regenerative Medicine Research, Institute of Biomedical Research and Innovation, Kobe, Japan.

^7^ Amakusa Hospital, Kumamoto, Japan.

^8^ Institute for Ageing and Health, NIHR Biomedical Research Building, Newcastle University, Campus for Ageing and Vitality, Newcastle upon Tyne, UK.

^9^ Department of Stroke and Cerebrovascular Diseases, National Cerebral and Cardiovascular Center, Osaka, Japan.

*Corresponding authors:

1. Masafumi Ihara, MD, PhD, FACP, Department of Stroke and Cerebrovascular Diseases, National Cerebral and Cardiovascular Center; 5-7-1 Fujishiro-dai, Suita, Osaka 565-8565, Japan; Phone: +81-6-6833-5012; FAX: +81-6-6835-5137; E-mail:ihara@ncvc.go.jp
2. Takakuni Maki, MD, PhD, Departments of Radiology and Neurology, Massachusetts General Hospital and Harvard Medical School; MGH East 149-2401, Charlestown, Massachusetts 02129, USA; Phone: +1-617-724-9503; FAX: +1-612-726-7830; E-mail: tmaki@partners.org

**Contents:**

**Supplemental Data, Material and Methods, References, Figures and Figure Legends, and Tables**

**Supplemental data**

***Cilostazol prevented the grooming performance in Tg-SwDI mice***

To evaluate cognitive function, we examined grooming performance by assessing the hair (ruffled coat) condition. Grooming performance has been reported to reflect cognitive function.^1, 2^ To assess grooming performance, the hair-coat condition was semi-quantitatively graded according to four categories (Supplemental Fig. 11A). Cilostazol-treated Tg-SwDI mice showed significantly better hair condition compared with vehicle-treated Tg-SwDI mice aged 15 months (cilostazol vs. vehicle; 0.4±0.1 vs. 1.4±0.4 for severity grade, *p*=0.014) (Supplemental Fig. 11B).

**Supplemental Material and Methods**

***Study approval***

Autopsied human brains were obtained from Kyoto University Hospital from 1992 to 2009 through a process approved by an institutional research committee. All animal procedures were performed according to the guidelines of the Animal Use and Care Committee of Kyoto University and of Institute of Biomedical Research and Innovation.

***Histological examination of postmortem human brains***

As described previously, the diagnosis of AD/CAA and non-AD/CAA was confirmed by clinical and pathological examinations.^3^ Briefly, the clinical diagnosis of dementia met the criteria of the Diagnostic and Statistical Manual of Mental Disorders IV. The neuropathological diagnoses of AD were made if the postmortem brains revealed the presence of frequent neuritic plaques in the neocortex (Consortium to Establish a Registry for Alzheimer’s Disease, CERAD), and NFT stage was no less than IV, according to the Braak and Braak neuropathological staging of Alzheimer-related changes.^3^ Tissue blocks were obtained from the frontal, temporal, parietal, and occipital lobes. The blocks were embedded in paraffin and sectioned at 12 μm thickness for Congo red staining, and 6 μm thickness for other staining on a microtome. Routine histological assessment was carried out with Congo red, hematoxylin and eosin (H&E), Klüver-Barrera, and modified Bielschowsky staining. Gallyas staining and thioflavin-S staining were added as required. The rest of the blocks were used for immunohistochemistry; this involved sequential incubation with primary antibody, followed by labeled polymer prepared by combining amino acid polymers with peroxidase and secondary antibody which is reduced to Fab' fragment (Nichirei Biosciences Inc.). The sections were rinsed with phosphate buffer saline (PBS) for 15 minutes between each step and finally visualized with 0.01% diaminobenzidine tetrahydrochloride and 0.005% H_2_O_2_ in 50 mM Tris-HCl (pH 7.6). The primary antibodies were mouse anti-Aβ protein antibody (1:50, DAKO), mouse anti-human paired helical filament-tau (AT8; 1:200, Thermo Scientific), and rabbit anti-PDE IIIA antibody (1:100, Abnova). Stage of CAA was divided into four grades proposed by Vonsattel, et al.^4^; ‘none’, no Aβ deposits in the vascular walls; ‘mild’, focal Aβ deposits in the smooth muscle layer of the vessel walls; ‘moderate’, circumferential Aβ deposits in the smooth muscle layer of the vessel walls; and ‘severe’, extensive Aβ deposition in the vasculature with morphological changes such as microaneurysms, fibrinoid necrosis, double barreling, inflammation, thrombus, or hemorrhage. When several grades were observed in one case, the dominant grade represented the case. The degree of PDE IIIA expression in the cerebrovasculature was classified into four grades; ‘none’, no expression; ‘low’, focal expression in the vascular wall; ‘medium’, circumferential expression in the vascular wall; and ‘high’, robust expression surrounding the entire vascular wall. PDE IIIA expression score was calculated from the average of qualified grading of PDE IIIA expression (0=none, 1=low, 2=medium, 3=high). From 17 patients, 1700 leptomeningeal and cortical arteries (100 per patient) were analyzed in the randomly selected 5 regions (20 vessels per one region) from the temporal and occipital lobes.

***Mice***

Tg-SwDI mice on a pure C57BL/6 background were obtained from The Jackson Laboratory. These mice express low levels of human Swedish/Dutch/Iowa mutant APP in neurons under the control of the mouse Thy1 promoter.^5^ Heterozygous Tg-SwDI and age-matched non-Tg C57BL/6 mice were used in the present study. The mice were fed with the pelleted chow containing 0.3% cilostazol (cilostazol-treated mice) or standard pelleted chow only (vehicle-treated mice). A previous study reported that the plasma cilostazol concentration in 0.1% cilostazol-treated rats was ~1 μM.^6^ Based on the *in vitro* IC_50_ value, this plasma cilostazol concentration is estimated to be sufficient to inhibit PDE III.^7^ In addition, previous reports have demonstrated that 0.1%-0.3% cilostazol-treated rats showed beneficial effects on vascular function.^7, 8^ Therefore, we used 0.3% cilostazol for treatment. The mice were housed in a room with a 12-hour light/dark cycle (lights on at 7:00 a.m.) with access to food and water *ad libitum*. The animals were assigned to three different groups as follows: (1) 12 months old at endpoint, treated from 4 months, (2) 15 months old at endpoint, treated from 1.5 months, and (3) 21 months old at endpoint, treated from 4 months. As Tg-SwDI mice develop Aβ deposits starting at 3 to 5 months of age,^5^ we evaluated the long-term effects of cilostazol by examining mice treated from the time point before and after Aβ deposits start. The animals were randomly assigned to cilostazol vs. vehicle, and assessments were done blinded to treatment group.

***Histological evaluation of PDE IIIA expression in mice***

After perfusion with PBS, brains were quickly snap-frozen. 10 μm-thick coronal sections were cut on cryostat at -20°C and placed on glass slides. After fixation and blocking, the sections were incubated with the anti-PDE IIIA primary antibody (1:100, Atlas Antibodies), followed by labeled polymer prepared by combining amino acid polymers with peroxidase and secondary antibody which is reduced to Fab' fragment (Nichirei Biosciences Inc.). The sections were rinsed with PBS for 15 minutes between each step and finally visualized with 0.01% diaminobenzidine tetrahydrochloride and 0.005% H_2_O_2_ in 50 mM Tris-HCl (pH 7.6).

***Measurement of cerebral blood flow***

Mice were anaesthetized with α-chloralose (50 mg/kg; i.p.) and urethane (750 mg/kg; i.p.). The trachea was intubated and mice were mechanically ventilated at a stoke volume of 5 ml/kg body weight and ventilation rate of 150 stokes/min with a ventilator. Rectal temperature was maintained between 36.5°C and 37.5°C. The stability of the level of anaesthesia was checked by testing corneal reflexes and motor responses to tail pinch. Relative cerebral blood flow (CBF) was determined by laser speckle perfusion imaging (Omegazone; Omegawave Inc.), which obtains high-resolution, two-dimensional imaging^9^ and has a linear relationship with absolute CBF values, as determined by the [^14^C]-iodoamphetamine technique.^10^ Calibration was carried out with a calibration reference device (Calibrator S/N 080715-5; Omegawave Inc.) before each test. The mean CBF was measured in identically sized regions of interest (circle 1 mm in diameter) located 1 mm posterior and 2 mm lateral from the bregma. The skull was exposed by a midline scalp incision, the scalp was reflected laterally, and kept open throughout the experiment. During the measurement of CBF, the surface was diffusely illuminated by 780 nm laser light. The scattered light was filtered and detected by a CCD camera positioned over the head. The filter detected only scattered light that had a perpendicular polarization to the incident laser light. The raw speckle images were used to compute speckle contrast, which corresponds to the number and velocity of moving red blood cells, approximating CBF. Signal processing was performed by the algorithm developed by Forrester et al.^11^ Color-coded blood flow images were obtained in high-resolution mode (639 × 480 pixels; 1 image/sec). The sample frequency was 60 Hz. One blood flow image was generated by averaging numbers obtained from 20 consecutive raw speckle images. The recordings were initiated after the examiner confirmed that CBF did not change over 1 minute, and the five recordings of blood flow image were averaged. During the measurement of CBF, blood pressure was measured by the tail cuff method (MK-2000ST; Muromachi Co.) and confirmed to be kept constant.

***Evaluation of vascular responses to hypercapnia and acetylcholine***

To induce hypercapnia, mice were ventilated with 5% CO_2_ for 5 minutes followed by ventilation with 20% O_2_ containing air. To evaluate vascular responses to vasodilatory stimuli, a cranial window preparation was performed as previously reported with modification.^12, 13^ Firstly, the CBF changes in response to hypercapnia were evaluated, with CBF values obtained every 30 seconds. The first five images were taken as baseline. Peak CBF increase during the 5 minutes after hypercapnia was taken as response amplitude. The rate of CBF increase after hypercapnia was calculated as the peak CBF increase (%) divided by the baseline CBF. Next, in order to assess the vasodilative responses, a 2 mm × 2 mm diameter craniotomy was performed with a dental drill in the right parietal bone. For real-time *in vivo* imaging of the cerebral vessels, we used fibered fluorescence microscopy (MVX10; Olympus). After intravenous tail vein injection of FITC dextran (2×10^6^ molecular weight, 200 μl of 20 mg/ml; Sigma), the leptomeningeal vessels were visualized. Ringer solution was infused into the cranial window. After the evaluation of vessel diameter response to hypercapnia, the dura was removed and the cranial window was subsequently infused with Ringer solution for 10 minutes until the vessel diameter returned to its baseline value. Then, the endothelium-dependent vasodilator acetylcholine (100 μM; Sigma) was infused into the cranial window at a rate of 100 μl/min for 5 minutes. The zoom, focus, gain values, and sampling time were kept constant during data acquisition in each section analyzed. The images were obtained every 1 minute and processed using Adobe Photoshop (version 7, Adobe Systems). Averaged vessel diameter across a 25 μm longitudinal segment (8 consecutive segments per mouse) of the dorsal middle cerebral arteries were analyzed before (baseline) and after inhalation of 5% CO_2_ or treatment with acetylcholine as previously described.^12^ Peak vessel diameter increase during the 5 minutes was taken as response amplitude. Data were calculated as the increase of vessel diameter (%) divided by the baseline vessel diameter. We distinguished penetrating arteries from bridging (collecting) veins by identifying the location and also by following the direction of flow from the pial surface.

***Evaluation of perivascular drainage after intracerebral injections of soluble fluorescent Aβ***

Mice were anaesthetized by intraperitoneal injections of sodium pentobarbital (50 mg/kg body weight). The scalp was shaved and local anaesthetic (Lignocaine 5%) was placed in the external auditory meati as mice were positioned in a stereotaxic frame. Soluble fluorescent Aβ_1-40_ (HiLyte Fluo 488-labeled; AnaSpec) was injected stereotactically into the striatum (0.98 mm anterior and 1.5 mm lateral from the bregma, 3.0 mm in depth from the dorsal surface of the brain) (Fig. 3A, 3B) as previously reported.^14-16^ Fluorescent soluble Aβ_1-40_ was injected at a concentration of 100 μM in a volume of 0.5 µl over a period of 2 minutes through a glass micropipette with the injection tip 20 µm (TransferTips; Eppendorf). After each injection, the micropipette was left *in situ* for 2 minutes to minimize reflux back along the injection tract. Mice were killed at 30 minutes after the surgery, by terminal anaesthesia and transcardially perfused with heparinized 0.9% saline followed by 4% paraformaldehyde in 0.1M phosphate buffer (PB) pH 7.4. Brains were removed and further fixed by immersion in 4% paraformaldehyde for 6–8 hours and placed in 30% sucrose in 0.1M PB (0.2%NaN_3_) for cryoprotection. The brains were trimmed to form coronal blocks 4 mm thick with the injection site in the center using a brain tissue matrix. Blocks were then frozen in OCT compound (Tissue-Tek; Sakura) and sectioned in a coronal plane (20 μm-thick) on a cryostat (CM1850; Leica). Every tenth section was collected on a gel-coated slide for histological examination and quantification studies. To evaluate perivascular drainage of Aβ_1-40_, assessment of the fluorescent Aβ_1-40_ in a coronal plane on the dorsal surface of the brain was performed. The averaged and maximal values of radial extensions of fluorescent Aβ_1-40_ in the leptomeningeal vessels from the site of injection were calculated.

***Immunofluorescence***

Serial cryostat sections of brain, 20 μm-thick, were stained by immunocytochemistry to identify tissue elements in the brains following injection of fluorescent Aβ_1-40_. Sections were incubated at 4°C for 24 hours with primary antibodies diluted in PBS and then washed extensively with PBS at room temperature. Sections were incubated with secondary fluorochrome-conjugated antibodies in PBS for 90 minutes at room temperature and again washed extensively. Primary antibodies used for immunofluorescent analysis were as follows: rat antibody to platelet endothelial cell adhesion molecule-1 (1:100, BD Pharmingen), rabbit antibody to laminin (1:100, Sigma), and mouse antibody to α-smooth muscle actin (1:200, Sigma). Secondary antibodies used were as follows: TRITC-conjugated chicken antibody to rat IgG (1:100, Santa Cruz), TRITC-conjugated chicken antibody to rabbit IgG (1:100, Santa Cruz), and TRITC-conjugated rabbit antibody to mouse IgG (1:100, Dako).

***Histological investigation of Aβ accumulation***

Mice were deeply anaesthetized with sodium pentobarbital (50 mg/kg, intraperitoneal) and perfused transcardially with 0.01 M PBS. The removed brains were post-fixed in 4% paraformaldehyde in 0.1 M PB, and embedded in paraffin. They were then sliced into 6

µm-thick sagittal sections at 1 mm lateral from the midline, and subjected to H&E and modified Bielschowsky staining. Immunohistochemical staining was performed as previously described. As primary antibodies, rabbit anti-IgG (1:500, N1508; DAKO), rabbit anti-Aβ_1–40_ (1:500, Calbiochem), rabbit anti-Aβ_1–42_ (1:500, Calbiochem), and mouse anti-Aβ protein (1:500, 6E10; Signet) were used. The densitometric analysis of Aβ was performed blindly to animal groups by setting regions of interest in the cerebral cortex (600 × 400 μm, 3 regions) and the hippocampus using the identical threshold in the Aβ (6E10)-immunostained sections.

***Western blotting***

Western blot analysis of C-terminal fragments (CTFs) of APP was performed using protein extracts from the brain tissue of vehicle- and cilostazol-treated Tg-SwDI mice aged 15 months. Hippocampal tissue was homogenized in RIPA buffer (PBS, 1% Igepal, 0.5% sodium deoxycholate and 0.1% SDS with protease and phosphatase inhibitors (Sigma)). Homogenates were centrifuged at 700 × g for 5 minutes at 4°C and the supernatant was collected. Samples were separated by SDS-PAGE on 4–20% gels (Mini-PROTEAN TGX Precast Gel, Bio-Rad) and then transferred to nitrocellulose membranes at 100 V for 20 minutes, using Tris-glycine transfer buffer with 20% methanol. The membranes were rinsed briefly in PBS and dried completely, before being added to boiling PBS for 5 minutes, and rinsed in Tris-buffered saline with Tween 20. After blocking in 10% fetal bovine serum for 60 min, the membranes were incubated with anti-amyloid precursor protein, C-terminal antibody (1:6000, Calbiochem) at 4°C overnight. Immunoblots were then incubated with horseradish peroxidase-conjugated secondary antibody (1:20,000) for 30 minutes at room temperature. Immunoblots were detected using Enhanced Chemiluminescence Western Blotting Detection reagent (Amersham).

***Perls-Stieda’s iron staining***

After sections were deparaffinized and hydrated to distilled water, they were immersed in the solution of equal amount of hydrochloric acid and potassium ferrocyanide, which was prepared immediately before use, for 30–40 minutes. After the sections were washed in water 3 times, they were counterstained with 0.1% nuclear fast red for 3–4 minutes. Subsequently, after the sections were rinsed in water, they were dehydrated through 80% and 2 changes of 100% alcohol, and cleared in xylene. Then, the slides were coverslipped with resinous mounting medium.

***Evans blue extravasation***

The 10-month-old vehicle-treated Tg-SwDI mice were examined. One hour after 1 ml of 4% Evans blue (EB; Nakalai Chemicals Ltd.) in normal saline was injected intraperitoneally, the animals were anesthetized and then perfused transcardially with PBS followed by FITC dextran (1 ml of 20 mg/ml). Subsequently, the brains and other organs were snap-frozen, sectioned sagittally into 20 μm-thick slices, and examined by fluorescence microscopy.

***Electron microscopy***

Vehicle and cilostazol-treated Tg-SwDI mice were examined by electron microscopy at age of 21 months. After mice were perfused transcardially with 0.9% saline followed by 4% paraformaldehyde and 2% glutaraldehyde in 0.1 M PB, brains were removed and consecutively sectioned at the hippocampal level at a thickness of 1 mm using a brain tissue matrix. Then, brain tissues were fixed by immersion in 4% paraformaldehyde and 2% glutaraldehyde in 0.1 M PB for 48 hours at 4°C, and washed in 0.1 M PB (5 × 10 minutes). Two pieces of brain tissues (approximately 1.5 mm^3^ from each hippocampus including the subiculum) were subsequently resected and postfixed with 1% osmium tetroxide for two hours. Thereafter, the fixed tissue samples were dehydrated, infiltrated, and embedded in epoxy-resin (Luveak 812; Nakalai Tesque) for transmission electron microscope (TEM) study. Semi-thin sections (0.7 μm-thick) were cut and stained with 1% toluidine blue. Ultrathin sections (80 nm-thick) of selected areas were prepared on an ultramicrotome (EM UC6; Leica) and collected on 200-mesh cooper grids. These sections were counterstained with 2% uranyl acetate and lead citrate solution. The vessels were examined throughout the sample with a TEM (H-7650; Hitachi). For quantitative analysis, the number of microvessels with abnormal vascular walls was counted among all microvessels throughout 0.75–1 mm^2^ areas in each animal.

***Y maze test***

The Y maze test was performed as described previously.^9^ The Y maze is a three-arm maze with equal angles between all arms that is used to evaluate working memory. Mice were initially placed within one arm and allowed to move in the maze freely. The sequence and number of arm entries were recorded for each mouse over an 8-minute period. The percentage of triads in which all three arms were represented (ABC, CAB, or BCA but not BAB) was recorded as an alternation to estimate short-term memory of the last arms entered. The total number of possible alternations is the number of arm entries minus two. Additionally, the number of arm entries serves as an indicator of spontaneous activity.

***Evaluation of grooming performance***

Coat condition was used as an index of grooming^1^ and evaluated on a subjective assessment scale of 0 (best) –3 (worst) by an investigator blind to the experimental condition. As the animals aged, they showed abnormal grooming behavior as is seen in demented patients whose personal hygiene and grooming are impaired.

***Primary neuronal cell culture***

Cortical neuronal cultures were prepared from 17-day-old Wistar rat embryos (Charles River Laboratories) using methods described earlier.^17^ Briefly, cortices were dissected and dissociated using papain dissociation system (Worthington Biochemical Corporation). Cells were plated on dishes coated with polyethylenimine in high-glucose DMEM containing 5% fetal bovine serum and 1% penicillin/streptomycin at a density of 4 × 10^5^ per cells/ml. At 24 hours after seeding, the medium was changed to Neurobasal medium containing 0.5 mM glutamine, 1% penicillin/streptomycin, and 2% B27 supplement. Cells were cultured at 37°C in a humidified chamber of 95% air and 5% CO_2_. Cultures were used for experiments 14 days after seeding.

***Aβ ELISA***

After treatment of cultured neurons with 1–40 μM of cilostazol for 48 hours, the concentration of Aβ40 and Aβ42 in the conditioned media was measured by ELISA (Human/Rat βAmyloid 40 ELISA Kit II, Human/Rat βAmyloid 42 ELISA Kit, High-Sensitive, Wako Pure Chemical Industries, Ltd.) according to the manufacture's protocol.

***WST assay***

Cell viability was assessed by WST reduction assay (Dojindo). This assay is based on the detection of dehydrogenase activity of viable cells. The cells were incubated with 10% WST solution for 1 hour at 37°C. Then the absorbance of the culture medium was measured with a microplate reader at a test wavelength of 450 nm and a reference wavelength of 630 nm.

***Statistical analysis***

All values are expressed as means ±SD unless stated otherwise. Statistical analysis was conducted using Student’s t test (for behavioral tests), Mann Whitney U test (for those other than behavioral tests), and ANOVA followed by post hoc Tukey test or Tukey-Kramer test (for *in vitro* experiments). The correlation between the CAA severity and the PDE IIIA expression score was analyzed by Spearman's rank correlation coefficient (*Rs*). Differences with a probability value of *p*<0.05 were considered to be statistically significant and *p*<0.1 just marginally significant.

**Supplemental References**

1. Etherton MR, Blaiss CA, Powell CM, Sudhof TC. Mouse neurexin-1alpha deletion causes correlated electrophysiological and behavioral changes consistent with cognitive impairments. Proc Natl Acad Sci U S A. 2009;106:17998-18003.

2. Vidal R, Barbeito AG, Miravalle L, Ghetti B. Cerebral amyloid angiopathy and parenchymal amyloid deposition in transgenic mice expressing the Danish mutant form of human BRI2. Brain Pathol. 2009;19:58-68.

3. Okamoto Y, Yamamoto T, Kalaria RN, et al. Cerebral hypoperfusion accelerates cerebral amyloid angiopathy and promotes cortical microinfarcts. Acta Neuropathol. 2012;123:381-394.

4. Vonsattel JP, Myers RH, Hedley-Whyte ET, Ropper AH, Bird ED, Richardson EP, Jr. Cerebral amyloid angiopathy without and with cerebral hemorrhages: a comparative histological study. Ann Neurol. 1991;30:637-649.

5. Davis J, Xu F, Deane R, et al. Early-onset and robust cerebral microvascular accumulation of amyloid beta-protein in transgenic mice expressing low levels of a vasculotropic Dutch/Iowa mutant form of amyloid beta-protein precursor. J Biol Chem. y2004;279:20296-20306.

6. Miyamoto N, Tanaka R, Shimura H, et al. Phosphodiesterase III inhibition promotes differentiation and survival of oligodendrocyte progenitors and enhances regeneration of ischemic white matter lesions in the adult mammalian brain. J Cereb Blood Flow Metab. 2010;30:299-310.

7. Oyama N, Yagita Y, Kawamura M, et al. Cilostazol, not aspirin, reduces ischemic brain injury via endothelial protection in spontaneously hypertensive rats. Stroke. 2011;42:2571-2577.

8. Fujita Y, Lin JX, Takahashi R, Tomimoto H. Cilostazol alleviates cerebral small-vessel pathology and white-matter lesions in stroke-prone spontaneously hypertensive rats. Brain Res. 2008;1203:170-176.

9. Maki T, Ihara M, Fujita Y, et al. Angiogenic and vasoprotective effects of adrenomedullin on prevention of cognitive decline after chronic cerebral hypoperfusion in mice. Stroke. 2011;42:1122-1128.

10. Ayata C, Dunn AK, Gursoy OY, Huang Z, Boas DA, Moskowitz MA. Laser speckle flowmetry for the study of cerebrovascular physiology in normal and ischemic mouse cortex. J Cereb Blood Flow Metab. 2004;24:744-755.

11. Forrester KR, Stewart C, Tulip J, Leonard C, Bray RC. Comparison of laser speckle and laser Doppler perfusion imaging: measurement in human skin and rabbit articular tissue. Med Biol Eng Comput. 2002;40:687-697.

12. Han BH, Zhou ML, Abousaleh F, et al. Cerebrovascular dysfunction in amyloid precursor protein transgenic mice: contribution of soluble and insoluble amyloid-beta peptide, partial restoration via gamma-secretase inhibition. J Neurosci. 2008;28:13542-13550.

13. Park L, Wang G, Zhou P, et al. Scavenger receptor CD36 is essential for the cerebrovascular oxidative stress and neurovascular dysfunction induced by amyloid-beta. Proc Natl Acad Sci U S A. 2011;108:5063-5068.

14. Carare RO, Bernardes-Silva M, Newman TA, et al. Solutes, but not cells, drain from the brain parenchyma along basement membranes of capillaries and arteries: significance for cerebral amyloid angiopathy and neuroimmunology. Neuropathol Appl Neurobiol. 2008;34:131-144.

15. Hawkes CA, Hartig W, Kacza J, et al. Perivascular drainage of solutes is impaired in the ageing mouse brain and in the presence of cerebral amyloid angiopathy. Acta Neuropathol. 2011;121:431-443.

16. Hawkes CA, Sullivan PM, Hands S, Weller RO, Nicoll JA, Carare RO. Disruption of arterial perivascular drainage of amyloid-beta from the brains of mice expressing the human APOE epsilon4 allele. PLoS One. 2012;7:e41636.

17. Terasaki Y, Sasaki T, Yagita Y, et al. Activation of NR2A receptors induces ischemic tolerance through CREB signaling. J Cereb Blood Flow Metab. 2010;30:1441-1449.

**Supplemental Figure 1.**

The correlation between the CAA severity and the PDE IIIA expression score demonstrates significant linear correlation (*Rs*=0.8272, *p*<0.001). PDE IIIA expression score was calculated from the average of qualified grading of PDE IIIA expression (0=none, 1=low, 2=medium, 3=high).

**Supplemental Figure 2.**

(A-E) Representative images of PDE IIIA immunostaining in patients with AD and CAA. PDE IIIA expression is only slightly observed in neurites in the cerebral cortex (A) and pyramidal neurons in the hippocampal CA3 regions (B) and dentate gyrus (C) whereas it is strong in the cortical vessels in patients with AD/CAA (D, E). Scale bar indicates 50 μm.

**Supplemental Figure 3.**

Representative images of the leptomeningeal arteries (A–D), the cerebral cortex (E–H), and the senile plaques (I–L), which were stained for Aβ (A, E, I), paired helical filament-tau (PHF-tau) (B, F, J), PDE IIIA (C, G, K), and Congo red (D, H, L) in patients with AD and CAA. Of note, some neurons with dystrophic neurites (J, dashed arrow) showed PDE IIIA expression (G, K), but neuropil threads (F, arrows) and amyloid cores (I and L, arrows) were not positive for PDE IIIA. Scale bar indicates 50 μm.

**Supplemental Figure 4.**

PDE IIIA expression is increased in the vascular wall in aged Tg-SwDI mouse. Representative images of PDE IIIA immnostaining in the leptomeningeal (A–C) and intracortical (D–F) arteries of wild type mouse aged 12 months (A, D), Tg-SwDI mouse aged 4 months (B, E), and Tg-SwDI mouse aged 23 months (C, F). Scale bar indicates 20 μm.

**Supplemental Figure 5.**

Thioflavin-S staining in the Tg-SwDI mouse aged 23 months. Scale bars indicate 100 μm in A–F, 1 mm in G, and 500 μm in H and I.

**Supplemental Figure 6.**

Representative images of vehicle- (A) and cilostazol-treated (B) Tg-SwDI mice showing where vessel diameter was quantified. An averaged vessel diameter across a 25 μm longitudinal segment (8 consecutive segments per mouse) was analyzed before (baseline) and after inhalation of 5% CO_2_ or treatment with acetylcholine. Scale bars indicate 50 μm.

**Supplemental Figure 7.**

Tg-SwDI mice exhibit robust accumulation of both vascular and parenchymal Aβ with predominance of Aβ40 over Aβ42 in the perivascular/vascular areas without apparent microhemorrhage or BBB leakage at 15 months of age. (A–D) Representative images of Aβ40 (A), Aβ42 (B), and Aβ (6E10) immunostaining (C, D), Perls-Stieda’s iron staining (E), and IgG staining (F) in Tg-SwDI mice aged 15 months. Scale bars indicate 50 μm in A and B, 20 μm in C and D, and 100 μm in E and F.

**Supplemental Figure 8.**

Representative macroscopic (A, B) and microscopic fluorescent (C, D) images of the brain (A, C) and kidney (B, D) in the Tg-SwDI mouse aged 10 months after Evans blue, followed by FITC dextran (green), injection. There were clear differences in the extravascular extravasation of Evans blue (shown in blue in A, B, and red in C, D) between the brain and kidney. Scale bars indicate 50 μm.

**Supplemental Figure 9.**

Electron microscopic images of vehicle-treated (A-D) and cilostazol-treated (E, F) Tg-SwDI mice aged 21 months. (A) Multiple vesicles in the cytoplasm of pericytes. (B) Multiple vesicles (+) and damaged intracellular organelles including mitochondria replaced by dark material (upper inset) in the pericytic cytoplasm. Multiple vacuoles (++) and thickening/alterations of basement membrane (lower inset). The regions marked by dashed squares are enlarged in the right upper and lower inset, respectively. (C) Thickening/alterations of basement membrane. The region marked by a dashed square is enlarged in the inset. (D) Two transversely sectioned microvessels were seen connected by a dysmorphic process. Multiple stages of autosomes, lysosomes, and damaged intracellular organelles with disruption of mitochondrial membrane and reduced cristae (arrows, right lower inset) were present (++). The regions marked by dashed squares are enlarged in the right upper and lower inset, respectively. (E, F) Almost intact capillaries were seen in cilostazol-treated Tg-SwDI mice. Arrows indicate tight junction of endothelial cells. Pericytic mitochondria were normal. The region marked by a dashed square is enlarged (F, inset). Scale bars indicate 1 μm. As, astrocyte; Ax, axon; BM, basement membrane; E, endothelial cell; En, nucleus of endothelial cell; Ly, lysosome; m, mitochondrion; P, pericyte; Pn, nucleus of pericyte.

**Supplemental Figure 10.**

(A) Histograms showing number of arm entries (left) and alternation behavior (right) in the Y maze test of vehicle-treated (n=10) and cilostazol-treated (n=10) wild type mice aged 12 months treated from 11 months of age. Error bars indicate SD.

**Supplemental Figure 11.**

Grooming performance in vehicle-treated and cilostazol-treated Tg-SwDI mice. (A) Images showing the hair (ruffled coat) conditions graded as four categories 0–3. (B) Histogram showing grading score of the hair (ruffled coat) condition of vehicle-treated (n=10) and cilostazol-treated (n=12) Tg-SwDI mice aged 15 months. Error bars indicate SEM. **p*<0.05 in vehicle-treated Tg-SwDI mice vs. cilostazol-treated Tg-SwDI mice.

**Supplemental Table 1.** Detailed neuropathological features and analyses of 17 patients with AD or non-AD.

| No. | Age | Sex | Clinical diagnosis | Cause of death | Neuropathological diagnosis | CERAD | NFT Braak Stage | CAA grade | Other neuropathological findings | PDEIIIA expression score |
| --- | --- | --- | --- | --- | --- | --- | --- | --- | --- | --- |
| 1 | 68 | M | Dementia | Pneumonia | AD | Definite AD | IV | Moderate | ― | 1.3 |
| 2 | 86 | M | Dementia | Asphyxia | AD | Definite AD | VI | Severe | ― | 1.8 |
| 3 | 78 | M | Lobar hemorrhage | Lobar hemorrhage | AD | Probable AD | ND | Severe | ― | 1.8 |
| 4 | 83 | M | Lobar hemorrhage | Lobar hemorrhage | AD | Probable AD | ND | Severe | ― | 1.7 |
| 5 | 67 | F | AD | Pneumonia | AD | Probable AD | VI | Severe | ― | 2.1 |
| 6 | 93 | F | AD | Pneumonia | AD | Probable AD | V | Mild | An old lacuna | 1.0 |
| 7 | 84 | F | VaD | Pneumonia | AD+VaD | Probable AD | VI | Moderate | ― | 0.7 |
| 8 | 86 | F | VaD | Pneumonia | SIVD | Possible AD | IV | Moderate | A cerebellar infarct | 1.1 |
| 9 | 76 | F | VaD | Ileus | SIVD | Normal | 0 | Moderate | Meningioma,  A cerebral infarct | 1.0 |
| 10 | 80 | F | VaD | Pneumonia | SIVD | Normal | I | Mild | multiple lacunas | 0.7 |
| 11 | 75 | M | CLL | CHF | ― | Possible AD | ND | Mild | ― | 0.3 |
| 12 | 71 | M | ALS | Respiratory failure | ALS | Normal | 0 | Mild | ― | 0.6 |
| 13 | 79 | M | ALS | Respiratory failure | ALS | Normal | 0 | No | ― | 0.3 |
| 14 | 67 | M | ALS | Respiratory failure | ALS | Normal | I | No | ― | 0.3 |
| 15 | 73 | M | Lobar hemorrhage | Lobar hemorrhage | Lobar hemorrhage | Possible AD | III | Severe | ― | 2.3 |
| 16 | 89 | M | Dilated cardioimyopathy | Pneumonia | Dilated cardiomyopathy | Normal | III | No | ― | 1.0 |
| 17 | 93 | M | Pneumonia | Pneumonia | ― | Normal | III | No | ― | 0.8 |

These patients were also divided into 4 groups according to the Vonsattel grade of CAA (4 patients without CAA, 4 patients each with mild or moderate, and 5 patients with severe CAA). The grading score of PDE III expression is calculated from the average of qualified grade (0=none, 1=low, 2=medium, 3=high). In total, 1700 leptomeningeal and cortical arteries (100 per patient) were analyzed in the randomly selected 5 regions (20 vessels per one region) from the temporal and occipital lobes. AD, Alzheimer's disease; ALS, amyotrophic lateral sclerosis; CAA, cerebral amyloid angiopathy; CERAD, Consortium to Establish a Registry for Alzheimer’s Disease; CHF, congestive heart failure; CLL, Chronic lymphocytic leukemia; NFT, neurofibrillary tangle; SIVD, subcortical ischemic vascular dementia; VaD, vascular dementia; ND, not determined.
